# *Guerrerostrongylus marginalis* n. sp. (Trichostrongyloidea: Heligmonellidae) from the Guianan arboreal mouse (*Oecomys auyantepui*) from French Guiana

**DOI:** 10.1051/parasite/2016009

**Published:** 2016-03-08

**Authors:** Jessica M. Weirich, François Catzeflis, F. Agustín Jiménez

**Affiliations:** 1 Department of Zoology, Southern Illinois University Carbondale IL 62901-6501 USA; 2 CNRS UMR 5554, Institut des Sciences de l’Évolution, Case Courrier 064, Université Montpellier Montpellier 34095 France

**Keywords:** *Guerrerostrongylus marginalis* n. sp., Trichostrongyloidea, Heligmosomoidea, Heligmonellidae, *Oecomys auyantepui*, French Guiana

## Abstract

Based on the number and arrangement of cuticular ridges and configuration of the dorsal ray, nematode specimens collected from the small intestine of eight Guianan arboreal mice, *Oecomys auyantepui* (Rodentia: Sigmodontinae), in French Guiana are herein described and characterized. *Guerrerostrongylus marginalis* n. sp. (Heligmosomoidea: Heligmonellidae) shows a synlophe consisting of more than 40 ridges and a unique bursal arrangement with ray 8 (externo-dorsal) extending to the edge of the bursal margin, and appearing more prominent than the dorsal ray. This bursal arrangement is common in members of *Hassalstrongylus* Durette-Desset, 1971, but uncommon in the other four species in *Guerrerostrongylus* Sutton & Durette-Desset, 1991. The placement of the new species in *Guerrerostrongylus* is based on the number and nature of cuticular ridges and the ray arrangement and symmetry of the caudal bursa. Diagnostic characteristics of *Guerrerostrongylus marginalis* n. sp. include the length of ray 8 relative to bursal margin, the relative size of the spicules and vestibule, and the number of eggs in the uterus. We propose an amendment to the generic diagnosis of *Guerrerostrongylus* to modify the characters of the long rays 6 (postero-lateral), rays 8 (externo-dorsal), and dorsal ray as diagnostic, since at least ray 6 appears to be short in two different species in the genus, namely *G*. *ulysi* Digiani, Notarnicola & Navone, 2012 and *G*. *marginalis* n. sp.

## Abstract (fr)

Les spécimens de Nématodes prélevés de l’intestin grêle de huit Souris arboricoles des Guyanes, *Oecomys auyantepui* (Rodentia : Sigmodontinae) collectés en Guyane française sont ici décrits et caractérisés sur la base du nombre et de la disposition des crêtes cuticulaires et de la configuration de la crête dorsale. *Guerrerostrongylus marginalis* n. sp. (Heligmosomoidea : Heligmonellidae) montre un synlophe constitué de plus de 40 nervures et un agencement de la bourse unique avec le rayon 8 (externo-dorsal) se prolongeant vers le bord de la marge de la bourse, et apparaissant plus important que le rayon dorsal. Cette disposition de la bourse est fréquente chez les membres de *Hassalstrongylus* Durette-Desset, 1971, mais rare chez les quatre autres espèces de *Guerrerostrongylus* Sutton & Durette-Desset, 1991. L’attribution de la nouvelle espèce à *Guerrerostrongylus* est basée sur le nombre et la nature des crêtes cuticulaires et l’agencement des rayons et la symétrie de la bourse caudale. Les caractéristiques diagnostiques de *Guerrerostrongylus marginalis* n. sp. comprennent la longueur du rayon 8 par rapport à la marge de la bourse, la taille relative des spicules et du vestibule et le nombre d’œufs dans l’utérus. Nous proposons un amendement à la diagnose générique de *Guerrerostrongylus* en modifiant les caractères des longs rayons 6 (postéro-latéraux), rayon 8 (externo-dorsaux) et rayons dorsaux comme diagnostics, puisque au moins le rayon 6 semble être court chez deux espèces différentes du genre, à savoir *G. ulysi* Digiani, Notarnicola & Navone, 2012 et *G. marginalis* n. sp.

## Introduction

Trichostrongyloidea is the richest superfamily of nematodes both in the number of genera and species [5, 6]. They infect the stomach and small intestine of all terrestrial vertebrates. Their classification and taxonomy are chiefly based on features of the caudal bursa and synlophe [6, 9, 10]. Trichostrongyles featuring a caudal bursa type of 2-2-1, oblique axis of orientation of ridges of synlophe, and tails devoid of a spine are typically assigned to Heligmonellidae. These nematodes are found in talpoid insectivores, lagomorphs, and rodents, and have a cosmopolitan distribution [6]. The combination of characters in the caudal bursa and the number and orientation of ridges in the synlophe are used in the identification of genera in this family. Among them, *Guerrerostrongylus* Sutton and Durette-Desset, 1991 was proposed to include species with a minimum of 40 longitudinal ridges (slender and slightly salient, less numerous toward anterior end); long dorsal ray and ray 6 (postero-lateral); and females with not bent tails, partially covered with an invaginated cuticle [20]. Species included in *Guerrerostrongylus* share several traits with species in *Hassalstrongylus* Durette-Desset, 1971: however, the larger number of ridges in the synlophe and the relatively long size of the dorsal ray of the former have acted as reliable characters [7, 18, 20]. *Guerrerostrongylus* includes four known species that infect sigmodontine and caviomorph rodents throughout the eastern half of South America. These include the type species *G. uruguayensis* Sutton and Durette-Desset, 1991, *G. zetta* (Travassos, 1937), *G. gomesae* Simões, dos Santos and Maldonado, 2012, and *G. ulysi* Digiani, Notarnicola, and Navone, 2012. *Guerrerostrongylus uruguayensis* is found in *Oligoryzomys flavescens* (Waterhouse) from Uruguay and *Akodon simulator* (Thomas) from Argentina [2, 20]. *Guerrerostrongylus zetta* (Travassos, 1937) is found in *Oligoryzomys nigripes* (Olfers), *Akodon cursor* Winge, *Cerradomys subflavus* (Wagner), *Euryoryzomys russatus* (Wagner), *Nectomys squamipes* (Brants), *Oligoryzomys eliurus* (Wagner) and the caviomorphs *Galea spixii* (Wagler) and *Thrichomys pachiurus* Wagner from Argentina and Brazil [3, 18, 19, 22]. *Guerrerostrongylus gomesae* Simões, dos Santos and Maldonado, 2012 is found in *O. mamorae* Thomas in southwestern Brazil [18], whereas *G. ulysi* infects *Sooretamys angouya* (Fischer) from northeastern Argentina [3].

Herein we present the description of a new species of nematode that combines characteristics of both *Hassalstrongylus* Durette-Desset, 1971 and *Guerrerostrongylus*, yet it is assigned to the latter based on the number of ridges in the synlophe and the relatively long dorsal ray featured by males. The species was collected from the small intestine of the Guianan arboreal mouse, *Oecomys auyantepui* Tate, an arboreal and graminivorous sigmodontine rodent that is found throughout the Guiana Shield; these rodents are considered medium-sized with an average adult body mass of 40 g [1, 23]. In French Guiana, *O. auyantepui* is known through 60 preserved specimens from a dozen localities (unpublished data), within the large continuous track of primary rainforests; most collected animals have been caught in traps tied to lianas 1.0–1.5 m above the ground. The present findings constitute the first record of any endoparasite in this species of rodent. This new taxon is the fifth species in the genus and the uniqueness of its characters merits an emendation to the diagnosis of the genus that builds up in the variation in the number of ridges in the synlophe and the appearance of the bursal rays.

## Materials and methods

Eight Guianan arboreal mice, *O. auyantepui*, were captured in primary forest between 6 and 25 June 2011 in the locality of Cacao, French Guiana (04°33′ N, 52°26′ W). The individuals were caught in four different locations along a transect of approximately 1500 m in the well-drained *Terra Firme* forests (non-inundated by flooded rivers). The transect went through ridgetops and hillsides of old secondary and primary forests spanning elevations from 110 to 200 m above sea level. Other species of non-volant mammals caught during June 2011 in syntopy with the studied *O*. *auyantepui* were *Didelphis marsupialis* L., *Marmosa demerarae* (Thomas), and *Philander opossum* (L.) (Didelphidae); *Hylaeamys megacephalus* (Fischer), *Neacomys paracou* Voss, Lunde, and Simmons, and *Rhipidomys nitela* (Thomas) (Sigmodontinae); *Proechimys cuvieri* Petter, *P. guyannensis* (E. Geoffroy), and *Mesomys hispidus* (Desmarest) (Echimyidae). These mammals were collected using wire-mesh BTS traps and Sherman traps baited with peanut butter and local fruits and were placed in trees at different heights between 1 and 2 m as well as on the ground. The mammals were handled following the ethical chart of the American Society of Mammalogists [17]. Gastrointestinal contents were preserved in 70% ethanol and transported to the laboratory to be examined for helminths. Preservation, clearing, and mounting of parasites followed Pritchard and Kruse [14]. All helminths were preserved in 70% ethanol and kept under refrigeration.

Voucher specimens and paratypes of *G. zetta* (CHIOC7447, 35589), *G. gomesae* (CHIOC35667), *Hassalstrongylus epsilon* (Travassos, 1937) (CHIOC31608 31882), and *H. luquei* Costa, Maldonado, Bóia, Lucio, and Simões, 2014 (CHIOC35928) were borrowed from the Coleção Helmintológica do Instituto Oswaldo Cruz, Rio de Janeiro, Brazil (CHIOC). Type specimens were deposited in the Collection Helminthologique du Muséum National d’Histoire Naturelle, Paris, France (MNHN), CHIOC, the Colección Nacional de Helmintos of the Universidad Nacional Autónoma de México, Mexico City (CNHE), and the Harold W. Manter Laboratory of Parasitology of the University of Nebraska, Lincoln, US (HWML).

Nematodes were cleared in lactophenol and mounted on temporary slides; all measurements are in micrometers. For each character, the range is given first, followed by the average, coefficient of variation, and sample size (when different from the number of specimens used in the description). All measurements of holotype, allotype, and paratypes are available at http://opensiuc.lib.siu.edu/zool_data/9/. Mammalian specimens used in the helminthological examinations are part of the holdings of the Muséum d’Histoire Naturelle de Genève, Switzerland (MHNG), and the Muséum National d’Histoire Naturelle, Paris, France (MNHN).

Genomic DNA was extracted, isolated, and purified from three vouchered nematodes following standard protocols [12, 16]. These aliquots were used as a template to amplify a fragment of the mitochondrial gene coding for the large ribosomal subunit RNA (*rrnL*); the primers and thermal profile used to complete the reactions, as well as the postamplification processing of these fragments, are identical to those described elsewhere [11, 16]. Published sequences of available herpetostrongyles, heligmosomoids, heligmonellids, and viannaids were downloaded from GenBank, aligned using Clustal Omega (http://www.ebi.ac.uk/Tools/msa/clustalo/) and analyzed for phylogenetic signal using Parsimony and Maximum Likelihood as optimality criteria in PAUP* v4.b10 [21]. For the latter, the GTR + G model of evolution – estimated with jModelTest [13] – was enforced. To test for branch support, 1,000 bootstrap replicates were performed using a heuristic search. The posterior probability of all branches was calculated using MrBayes v3.2.5 [15], which ran for 10 million generations with resampling every 1,000 iterations for a final burn-in of 25%. The remaining trees were used to reconstruct the consensus. The matrix including the alignment and command lines used in both approaches is available at (http://opensiuc.lib.siu.edu/zool_data/8/).

## Results

### *Guerrerostrongylus* Sutton and Durette-Desset, 1991

Heligmonellidae. Medium-sized worms, with females reaching or exceeding 8 mm. Synlophe with at least 40 continuous cuticular ridges at midbody, sporadically 35 in males. Height of ridges in anterior half of the body unequal, height of ridges of similar size in posterior half. Caudal bursa sub-symmetrical, with ample dorsal lobe; ray 6 (postero-lateral) projected posteriad, dorsal ray long; ray 8 (externo-dorsal) usually shorter than dorsal ray. Bursal pattern of type 2-2-1 or 2-2-1 tending to 1-3-1. Genital cone not enlarged. Posterior end of female not bent; vulva opens near posterior end, tail tapers to a blunt end.

Type species: *Guerrerostrongylus uruguayensis.*

Other species: *Guerrerostrongylus zetta*, *G. gomesae*, *and G. ulysi*.

Hosts: Caviidae, Cricetidae, Echimyidae.

Site of infection: Small intestine.

Biogeographic region: Neotropics (Argentina, Brazil, French Guiana, Uruguay).

### *Guerrerostrongylus marginalis* n. sp. (Figs. 1–12)

urn:lsid:zoobank.org:act:4E636892-FA72-4894-9812-0E92610F4AB8

Type host: Guianan arboreal mouse, *Oecomys auyantepui* Tate, 1939 (Cricetidae: Sigmodontinae). Symbiotype [8]: field number V-2934 collected on 09 June 2011 near Cacao, French Guiana, MHNG-1979.066.

Other hosts: *Hylaeamys megacephalus* (Fischer).

Type locality: France: French Guiana: Cacao: (Municipality of Roura): 04°33′708 N; 52°26′590 W; altitude 197 m.

Prevalence, mean, and range of intensity: 100%, 35, 4–132. One worm in *H. megacephalus*.

Site of infection: Small intestine.

Specimens deposited: Holotype and allotype MNHN 89YT, paratypes MNHN 90YT, 91YT, 92YT; CHIOC 38104–05, HWML 91932–34, CNHE9092.

Etymology: The species name, *marginalis*, refers to the extension of ray 8 (externo-dorsal), which reaches the posterior margin of the bursa.

#### Description

**Figures 1–6.**
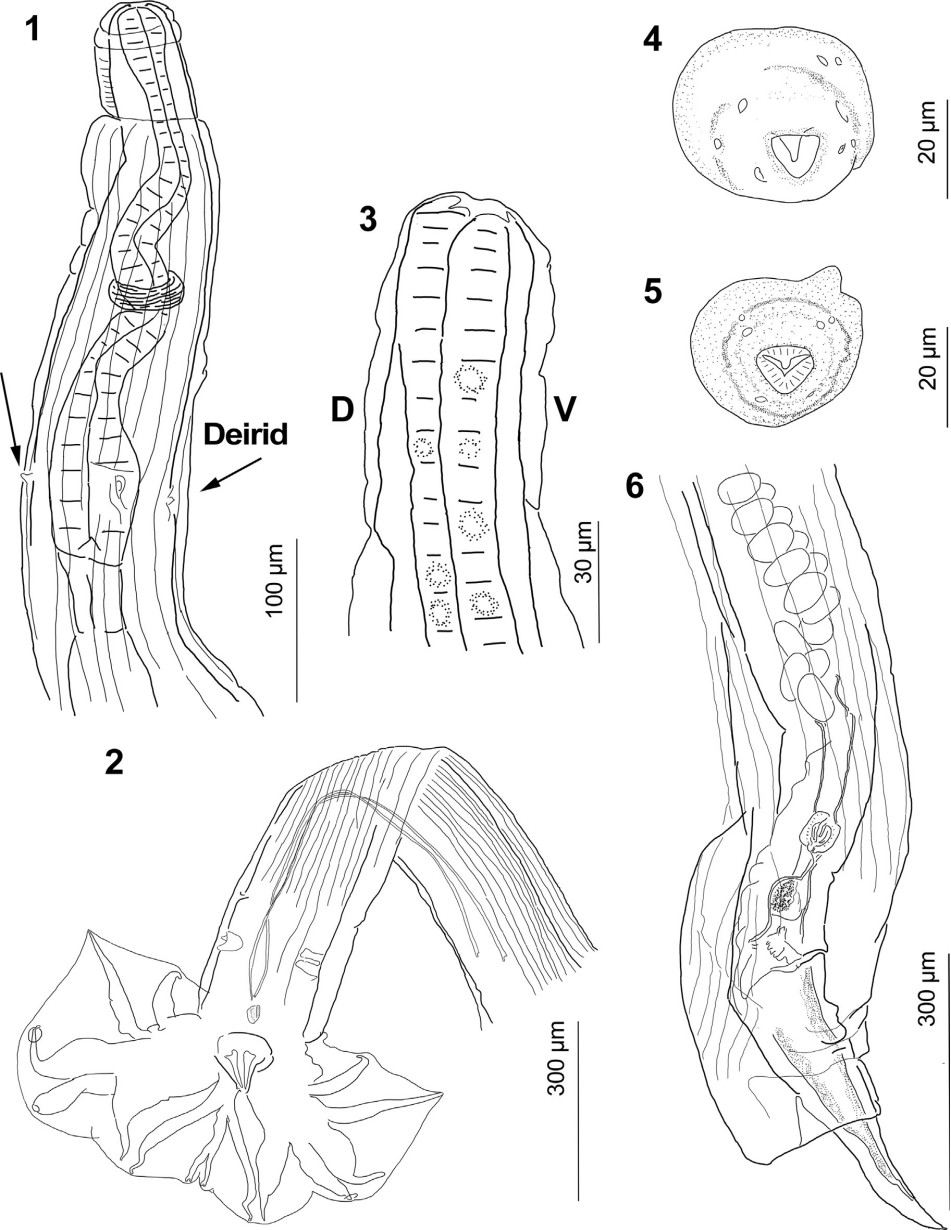
*Guerrerostrongylus marginalis* n. sp. **1,** Ventral view of the anterior end of male, showing cephalic vesicle, esophagus, nerve ring, deirids (indicated by arrows), and excretory pore (between deirids). **2,** Posterior end of a paratype, showing caudal bursa, genital cone, and spicules. **3,** Lateral view of cephalic vesicle and stoma with esophageal tooth (upper left) not projected toward lumen. **4,** Apical view of a female featuring dorsal tooth and triangular stoma. **5,** Apical view of a male, showing dorsal tooth and triangular stoma. **6,** Posterior end of a paratype showing cuticular invagination covering vulva, vulva, anus, ovejector, infundibulum, eggs in uterus, and tail.

General: Slender, medium-sized nematodes. Sexually dimorphic, body slightly coiled, females larger than males. Well-developed cephalic vesicle (Figs. 1, 3). Stoma triangular, dorsal esophageal tooth not projected toward lumen (Figs. 3–5), two amphids and four submedian cephalic papillae, only two externolabial papillae were observed in both male and female (Figs. 4, 5).

**Figures 7–12.**
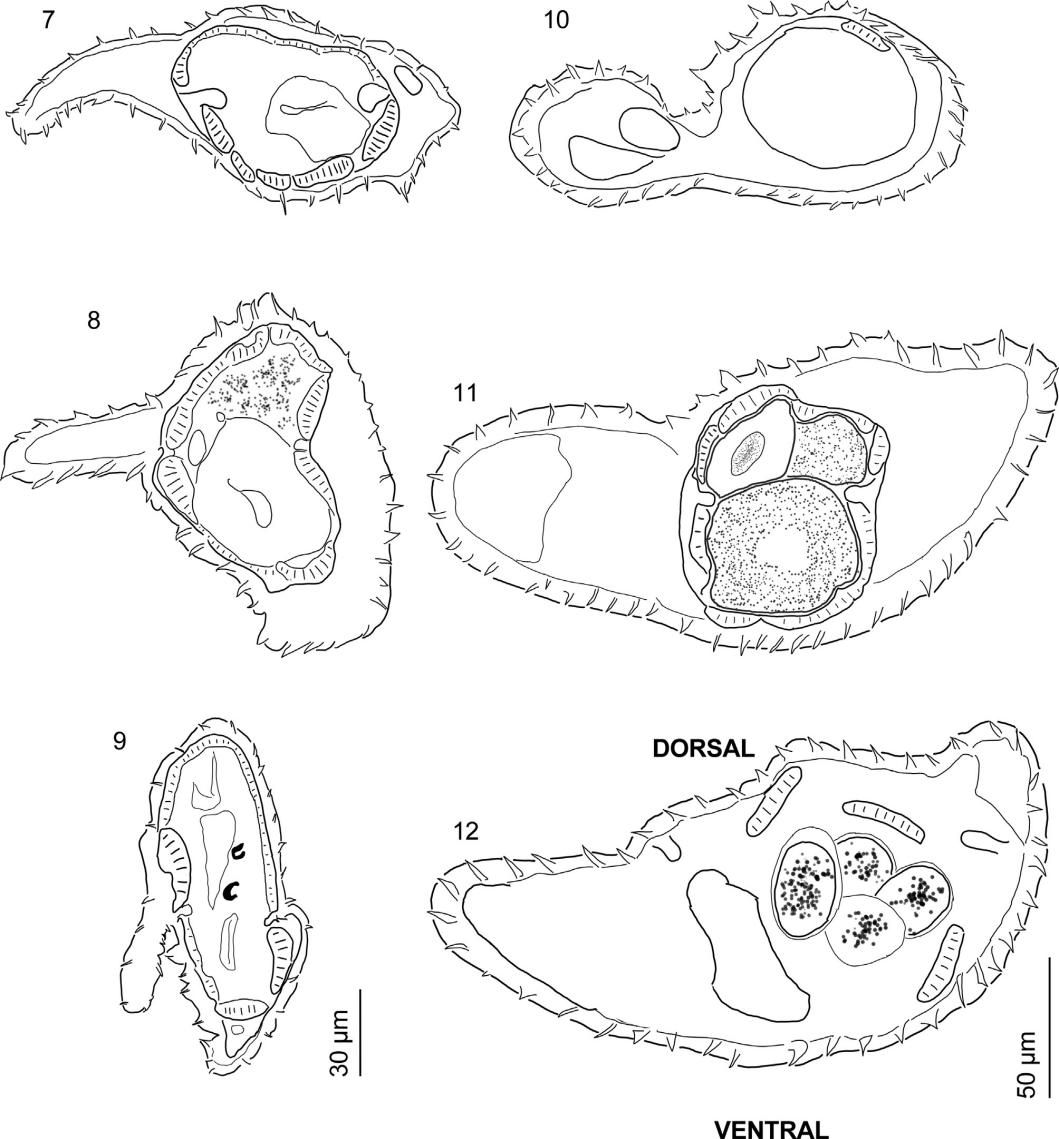
*Guerrerostrongylus marginalis* n. sp., orientation of all sections is dorsal side toward the top of page, ventral side toward the bottom of page. **7–9**, Synlophe of male paratype, scale bar 30 μm. **7,** At level of esophagus. **8,** At midbody. **9,** At posterior end, showing spicules. 9–11 Synlophe of female paratype, scale bar = 50 μm. **10,** At level of esophagus. **11,** At midbody. **12,** At proximal portion of uterus.

*Synlophe* (*based on 5 males and 7 females*): With continuous ridges, beginning just posterior to cephalic vesicle ending immediately anterior to vulva and bursa. Ventral and dorsal ridges straight, lateral ridges converge in space between deirids and cephalic vesicle. Left ridges slightly smaller than rest, especially in anterior half; orientation of ridges subfrontal, ridges on ventro-dextral and dorsodextral quadrants oriented to the left. Ridges more numerous at midbody. At level of esophagus, males feature 37–39 ridges (Fig. 7) and females 36–46 ridges (Fig. 10); at midbody, males feature 36–45 ridges (*n* = 3; Fig. 8) and females 36–45 ridges (Fig. 11). Finally, males feature 34–44 ridges at level of spicules (*n* = 4; Fig. 9) and females 25–45 ridges at level of distal uterus (Fig. 12).

*Male*: (*measurements based on 25 specimens*, *unless otherwise noted*): Body length 4,156–6,741 (5,437, 15%, *n* = 23), width at midbody 151–266 (203, 20%, *n* = 23); cephalic vesicle 44–89 (70, 14%) long and 33–74 (46, 17%) wide; excretory pore, deirids, and nerve ring situated at 174–388 (269, 24%, *n* = 13), 178–393 (253, 26%, *n* = 9), and 139–282 (187, 29%, *n* = 6) from anterior end, respectively; esophagus 320–419 (361, 8%, *n* = 16) long, 23–54 (32, 25%, *n* = 15) wide (Fig. 1). Caudal bursa sub-symmetrical, with right lobe slightly larger, dorsal lobe with cleft, ray pattern 2-2-1 tending to 1-3-1. Ray 2 directed anteriad, curved medially. Ray 3 longer than ray 2, straight, reaching bursal margin (Fig. 2). Ray 4 slightly longer than ray 5, both divergent, ray 4 directed anteriad, ray 5 slightly curved posteriad. Ray 6 directed posteriad, not reaching bursal margin. Ray 8 arising from proximal quarter of dorsal ray, reaching bursal margin. Dorsal ray long, divided at about distal quarter into two branches, each bifurcates into rays 9 (external branches) and rays 10 (internal branches). Conspicuous genital cone 51–91 (65, 15%) long, 34–95 (67, 20%, *n* = 24). Spicules thin, subequal, right spicule 544–829 (687, 12%, *n* = 22) long, 5–11 (8, 18%, *n* = 22) width; left spicule 545–825 (686, 12%, *n* = 21), 6–13 (8, 23%, *n* = 21). Gubernaculum 21–39 (28, 16%, *n* = 24) long, 13–24 (19, 14%, *n* = 21) wide (Fig. 2).

*Female* (*measurements based on 35 specimens*, *unless otherwise noted*): Body length 5,070–12,417 (8,635, 23%), width at posterior end 129–432 (261, 28%); cephalic vesicle 50–97 (73, 15%, *n* = 33) long and 38–89 (50, 20%, *n* = 33) wide; excretory pore, deirids, and nerve ring situated at 156–389 (263, 21%, *n* = 25), 207–402 (275, 17%, *n* = 15), 154–254 (178, 23%, *n* = 6) from anterior end, respectively. Esophagus 303–468 (381, 13%, *n* = 27) long, 29–75 (40, 24%, *n* = 23) wide. Monodelphic. Vulva 232–466 (342, 18%) from caudal end; short vagina 38–88 (50, 20%, *n* = 32), connected to vestibule 91–205 (138, 17%) long and 35–82 (59, 22%) wide; sphincter 22–70 (34, 35%) long, 16–74 (28, 48%) wide, connected to infundibulum 44–259 (153, 30%, *n* = 33) (Fig. 6). Uterus 1,114–2,020 (1,507, 17%, *n* = 10), containing 70–201 eggs (110, 39%, *n* = 11). Eggs 50–72 (59, 8%, *n* = 216) long by 30–60 (36, 11%, *n* = 216) wide. Tail conical, not curved. Distance from cuticular invagination and anus to distal end 141–356 (233, 21%, *n* = 33), and 50–86 (62, 15%, *n* = 28), respectively.

#### Differential diagnosis

*Guerrerostrongylus marginalis* is different from the other four species in the genus in the extension of ray 8 relative to rays 9 and 10. In *G. marginalis*, ray 8 extends more posteriorly than rays 9 and 10, yet all reach the posterior margin of the bursa; in all other species, ray 8 appears to be shorter than rays 9 and 10, and consequently, rays 8 do not reach the posterior margin of the bursa. Also, the length of the dorsal ray in *G. marginalis* represents 50% of the length of the caudal bursa, whereas in most of the species in the genus this proportion is greater than 60%. This characteristic makes the dorsal lobe to appear “long” relative to the length of the bursa. In addition, both dorsal ray and ray 6 of *G. marginalis* appear to be proportionally shorter than rays 3–5 and therefore, to the caudal bursa.

Other characters that assist in the discrimination of *G. marginalis* from other species in the genus include a combination of the relative size of the spicules, size of genital cone, length of the uterus, and size of eggs (Table 1). A comparison against each species follows. First, *G. ulysi* features a proportionally longer dorsal ray that causes rays 9 and 10 to extend farther posteriad than rays 6 and 8; in *G. ulysi* the length of the dorsal ray represents 60% of the length of the caudal bursa. Second, *G. marginalis* can be discriminated from *G. zetta* in the relative length of rays 6 and 8, in addition, the dorsal ray is 70% of the length of the caudal bursa. Regarding traits in females, the vulva in *G. zetta* appears to be closer to the posterior end than the vulva of *G. marginalis*. Third, the dorsal ray in *G. uruguayensis* is 65% the length of the caudal bursa; in contrast, the genital cone is very small in *G*. *uruguayensis* (14 × 9 versus 71 × 72 in *G. marginalis*). Interestingly, both uterus and vestibule are longer in *G. uruguayensis* (2,500 and 350, respectively) than the homologous structures in *G. marginalis* (2,020 and 205, respectively). Finally, the most similar species to *G. marginalis* is *G. gomesae*, yet both can be discriminated because ray 5 of *G*. *gomesae* appears to be relatively longer than ray 6. In contrast, the spicules as well as the eggs of *G. marginalis* tend to be larger. The range for spicules is 544–829 (average 717) for *G. marginalis* and 310–560 for *G. gomesae*, whereas the range for their eggs is 31–59 × 25–35 and 50–72 × 30–60, respectively. The number of eggs in the uterus of *G. marginalis* is greater than the number of eggs in *G*. *gomesae*. Another notable difference is the length of the vestibule, which is reportedly shorter in *G*. *gomesae* than the homologous structure in *G. marginalis* (Table 1).

**Table 1.** Comparative measurements of diagnostic traits for males in *Guerrerostrongylus* Sutton and Durette-Desset, 1991. For *G. marginalis* the range is followed by measurements of the type. Values in parentheses include structures measured in three paratypes of *G. gomesae*. All measurements are in μm.

	*G. marginalis* present work	*G. uruguayensis* Sutton & Durette-Desset, 1991	*G. gomesae* Simões, dos Santos, Maldonado, 2012	*G. ulysi* Digiani, Notarnicola, Navone, 2012	*G. zetta* (Travassos, 1937)	*G. zetta* paratypes (Simões et al. 2012)	*G. zetta* Argentina (Digiani et al. 2012)
**Males**	**Range, holotype**						
Body length	4,156–6,741, 6,181	9,150	4,524–7,240	5,350–8,320	6,400	4,280–6,900	4,400–8,400
Maximum width	151–266, 195	275	150–210	130–180	150	80–180	140–290
No. of ridges in midbody synlophe	36–44	40–45	35–46	42–44		36–42	40–44
Cephalic vesicle							
Length	44–89, 65	60	40–61	60–72	45–52	40–73	35–70
Width	33–74, 40	60	30–60	40–50		20–56	30–60
Distance from anterior end to:							
Nerve ring	174–388, 176	210	147–177	165–240		70–233	190–295
Excretory pore	139–282, 233	325	300–310	250–340	200	229–633	250–345
Deirids	178–393, 239	340	300–360	280–330			250–381
Esophagus length	320–419, 338	500	310–360	380–450	340–470	340–716	345–495
Corpus width	23–54, 23				37–45		
Right spicule	544–829, 717	1,110	310–560	455–665	877	580–1,160	750–1,420
Left spicule	545–825, 735						
Gubernaculum							
Length	21–39, 28	19.6	10–30	25–32		21–47	28–40
Width	13–24, 18	10	10–21	15–15		10–20	15–25
Genital cone							
Length	51–91, 71	14.5	45–93	40–65		40–70	
Width	34–95, 72	9	43–63	35–45		20–66	
**Females**	**Range, allotype**						
Body length	5.070–12,417; 6,961	12,900	6,700–8,440	5,500–13,100	6,800–7,300	5060–12,670	5,500–13,700
Maximum width	129–432, 228	300	140–320	110–250	140–150	100–320	100–290
No. of ridges in midbody synlophe	36–45	44–46	40–48	40–46		38–42	35–48
Cephalic vesicle							
Length	50–97, 74	75	37–62	50–72		40–74	35–65
Width	38–89, 48	40	33–52	35–50		36–67	35–60
Distance from anterior end to:							
Nerve ring	154–254, 154	240	160–210	145–200		100–250	130–285
Excretory pore	156–389, 298	380	300–403	235–310		221–402	345–380
Deirids	207–402, 307	400	310–350	245–320			235–275
Esophagus length	303–468, 378	580	318–415	370–550		292–437	350–500
Corpus width’	29–75, 38						
Distance vulva – Posterior end	232–466, 306	200	250–422	110–250	112–135	105–233	112–255
Vagina	38–88, 36	25	32–47 (26–35)	15–30		23–60	15–25
Vestibule length	91–205, 158	350	27–50 (55–63)	100–160	210–225	170–230	160–310
Vestibule width	35–82, 60		(18–35)				
Sphincter length	22–70, 42	80	8–14 (28–40)	40–50		50–60	40–65
Sphincter width	16–74, 38	65	38–45 (23–31)	40–50		33–63	40–80
Infundibulum	44–259, 197	300	22–41 (210–300)	160–240		150–340	160–315
Uterus	1,114–2,020; 1,498	2,500	906–1,363	600–2,800		1,350–2,540	800–1,560
Eggs *in utero*	70–201, 74	+200	48, 62	20–170		–	6–50
Eggs’ length	50–72		31–59	65–80	56–63	60–70	55–72
Eggs’ width	30–60		25–35	38–50	25–30	30–40	38–45
Tail	50–86, 74	75	45–78	40–50	43–45	43–97	40–100

#### Molecular results

**Figure 13.**
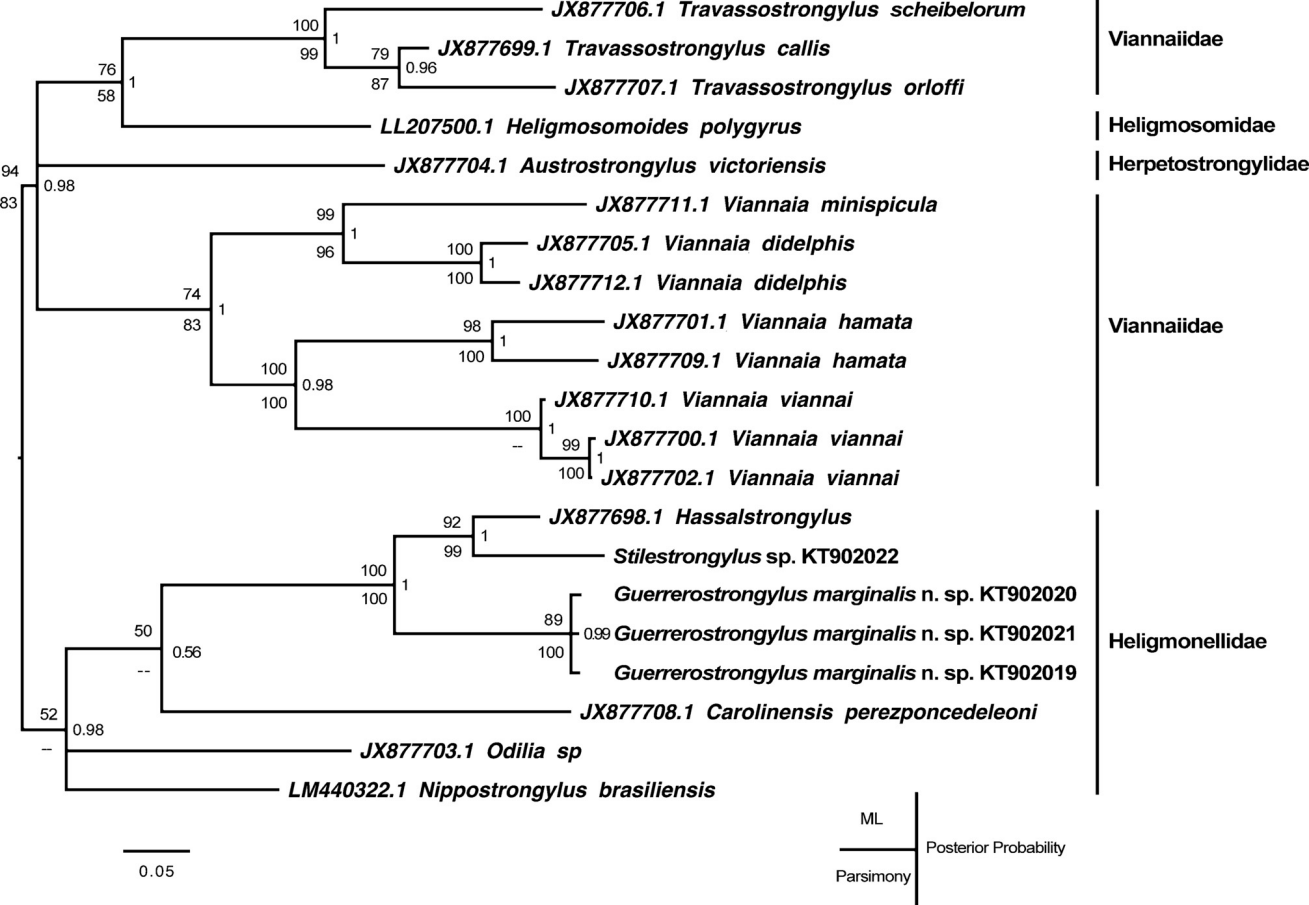
Placement of *Guerrerostrongylus marginalis* n. sp., relative to available heligmonellid nematodes. The phylogenetic tree (based on a fragment of the mitochondrial gene coding for the large ribosomal subunit RNA – *rrnL*-) represents the consensus used to calculate the posterior probability of the branches. Posterior probability is to the right of each node. Bootstrap support values are indicated to the left of the node, with values for Maximum Likelihood support over those obtained by parsimony.

The phylogenetic reconstruction based on the mitochondrial gene *rrnL* is shown in Figure 13. This tree is the consensus resulting from the estimation of the posterior probabilities of the branches. The analysis of the dataset using parsimony and Maximum Likelihood results in six and three trees, respectively. The trees obtained using Maximum Likelihood are essentially the same, since the only difference is the reciprocal position of the specimens identified as *G. marginalis*. The six trees generated with parsimony as optimality criterion have a length of 1,411 steps and a consistency index of 0.43, resulting from the analysis of 347 parsimony informative characters; these trees vary in the position of *Nippostrongylus brasiliensis*, *Heligmosomoides polygyrus*, and *Austrostrongylus victoriensis*, relative to species of *Travassostrongylus* and *Viannaia*. Nevertheless, the monophyly of *G. marginalis* is supported in all three analyses (Fig. 12). This species appears to be clustered with the heligmonellid *Hassalstrongylus* sp. and *Stilestrongylus* sp., in a clade that shows a strong support of 100% and a posterior probability of 1.

## Discussion

The configuration of the caudal bursa of *G. marginalis* resembles the homologous structure in some species of *Hassalstrongylus.* This is because the extension of the dorsal ray appears to be 50% the length of the caudal bursa, ray 8 extends more posteriad than rays 9 and 10, and the extension of rays 4 through 6 gives the caudal bursa the appearance of an irregular trapezoid. The perception of the overall shape of the caudal bursa of *G. marginalis* seems to differ from the caudal bursa of other members of *Guerrerostrongylus*, which was described as ellipsoidal, rectangular, or heart-shaped [3, 18]. Irrespective of the interpretation of the shape of the bursa, the overall symmetry in all five species is sub-symmetrical as described in Durette-Desset and Digiani [7]. Additionally, the number of ridges in the synlophe, the size variation of these ridges, and the posterior end of the females are typical of *Guerrerostrongylus*.

The original diagnosis of the genus was based on two species that bear striking morphological resemblances, namely *G. uruguayensis* and *G. zetta*. Since its original description [20], the diagnosis has been translated into English [9], yet this diagnosis predates the description of three more species (*G. gomesae*, *G. ulysi*, and *G. marginalis*) that show more variability in some of the characters used for the diagnosis, including the size of the worms, the number of ridges in the synlophe, and the relative size of rays 6, 8, and dorsal (including rays 9 and 10). For example, ray 6 in *G. ulysi* is not as long as the homologous structure in *G. uruguayensis* and *G. zetta.* Furthermore, the number of ridges at midbody in the synlophe of males of *G. gomesae* can be 36 [18], which is also the case for *G. marginalis*. Although the proposed changes are minor, the emended diagnosis we present accounts for the variability observed in the number of ridges and the sub-symmetrical shape of the caudal bursa. The direct observation of paratypes of *G. gomesae* allows the detection of minor inconsistencies in the measurements of the vagina, vestibule, and sphincters. The range for these measurements is noted in parentheses in Table 1, and it also includes the number of eggs counted in the uteri of two paratypes. Digiani et al. [4] have shown that this value, as well as the length of the uterus, are reliable characters to assist in the discrimination of syntopic species of *Hassalstrongylus*. This suggests that the statistical analyses of meristic data may yield unexpected useful characters in species discrimination. With the expectation that other scientists can complete these tests, we have made the measurements for the type specimens universally available (http://opensiuc.lib.siu.edu/zool_data/9).

For the completion of the present work, specimens of *G. zetta* collected from *Oligoryzomys nigripes* (Olfers) in Argentina were kindly provided by Dr. Mike Kinsella. Unfortunately, attempts to amplify DNA from these individuals failed, perhaps as a result of their previous contact with clearing reagents. As a consequence, the relationship of *G. marginalis* with the rest of the species, as well as their placement in Heligmonellidae, remains to be tested.
